# A Talk-Listen-Ack Beaconing Strategy for Neighbor Discovery Protocols in Wireless Sensor Networks

**DOI:** 10.3390/s22010377

**Published:** 2022-01-04

**Authors:** Zhong Shen, Yongkun Yao, Kun Zhu, Xin Xiang

**Affiliations:** 1 State Key Laboratory of Integrated Service Network, Xidian University, Xi’an 710071, China; 2 School of Telecommunications Engineering, Xidian University, Xi’an 710071, China; ykyao@stu.xidian.edu.cn (Y.Y.); zhukun@stu.xidian.edu.cn (K.Z.); 3 Guangzhou Institute of Technology, Xidian University, Guangzhou 510555, China; xiangxin@stu.xidian.edu.cn

**Keywords:** wireless sensor networks, neighbor discovery, beaconing, duty cycle

## Abstract

Neighbor discovery is a fundamental function for sensor networking. Sensor nodes discover each other by sending and receiving beacons. Although many time-slotted neighbor discovery protocols (NDPs) have been proposed, the theoretical discovery latency is measured by the number of time slots rather than the unit of time. Generally, the actual discovery latency of a NDP is proportional to its theoretical discovery latency and slot length, and inversely proportional to the discovery probability. Therefore, it is desired to increase discovery probability while reducing slot length. This task, however, is challenging because the slot length and the discovery probability are two conflicting factors, and they mainly depend on the beaconing strategy used. In this paper, we propose a new beaconing strategy, called talk-listen-ack beaconing (TLA). We analyze the discovery probability of TLA by using a fine-grained slot model. Further, we also analyze the discovery probability of TLA that uses random backoff mechanism to avoid persistent collisions. Simulation and experimental results show that, compared with the 2-Beacon approach that has been widely used in time-slotted NDPs, TLA can achieve a high discovery probability even in a short time slot. TLA is a generic beaconing strategy that can be applied to different slotted NDPs to reduce their discovery latency.

## 1. Introduction

Recent years have witnessed a large variety applications of wireless sensor networks in industrial Internet of Things, environmental monitoring and protection, smart agriculture, and smart city. In all these applications, neighbor discovery [1] is a fundamental function for sensor networking, because the knowledge of neighboring nodes is the prerequisite for link establishment, routing, and network communication.

Sensor nodes discover each other by sending and receiving beacons. Sensor nodes are typically low-cost and battery-powered devices. They cannot always turn on the radio to communicate with other nodes, because the battery cannot be replaced in most applications. Generally, sensor nodes work in a low-power mode: they turn off the radio and go to sleep most of the time; only when needed or according to scheduling, they turn on the radio for a while to communicate. The percentage of the time that a node’s radio is on is called its duty cycle. As the duty cycle of a node decreases, the chance of discovering other nodes and being discovered by other nodes becomes lower. Furthermore, sensor nodes may be mobile devices and do not have time synchronization information. Therefore, the neighbor discovery protocol (NDP) designed for wireless sensor networks should take duty cycle, mobility, and asynchronous information into account [1].

In many applications, it is desirable for neighbor discovery to achieve both low duty cycle and low discovery delay. For example, in habitat monitoring applications, sensors are carried by animals for long-term research, and neighbor discovery enables sensors to record activity and exchange data even during short periods of contact, which helps scientists understand their interactions and mutual influences. In manufacturing asset tracking applications, by attaching sensors to equipment and deploying sensors in the manufacturing area, neighbor discovery enables the management system to track the location of equipment in real time throughout the manufacturing process. In health care monitoring applications, through the sensors worn by students, neighbor discovery can collect all contacts within the transmission distance of infectious diseases in a timely manner, so as to model the disease propagation and contain infectious diseases [2].

Many energy-efficient neighbor discovery protocols are proposed for wireless sensor networks. These NDPs can be divided into two categories: probabilistic and deterministic. As a representative of probabilistic NDPs, Birthday protocol [3] transmits, receives, or sleeps with a particular probability in each time slot. The average discovery latency of Birthday is low, but the worst-case latency is not bounded. In contrast, deterministic NDPs such as Disco [4], U-Connect [5], Searchlight [6], Hello [7], and Nihao [8] can guarantee that the worst-case latency is bounded. The design techniques used by most existing deterministic NDPs include coprime (e.g., Disco [4]), quorum (e.g., Searchlight [6] and Nihao [8]), or hybrid of both (e.g., U-Connect [5] and Hello [7]). These techniques ensure that any two neighboring nodes have overlapping time within the worst-case latency to send/receive beacons to each other.

Time-slotted model are widely used by existing NDPs [3,4,5,6,7,8,9,10,11], in which time is divided into fixed-length slots. Each NDP has its own cycle, which consists of a certain number of time slots, some of which are selected as working slots (or active slots), and other time slots are selected as non-working slots (or sleeping slots). During an active slot, a node will turn on its radio to transmit/receive beacons.

Although many time-slotted NDPs are proposed, there are still some issues. First, the discovery latency of a time-slotted NDP is measured by the number of time slots rather than the unit of time [12]. Second, in most slotted NDPs, the 2-Beacon approach that beacons are sent at the beginning and the end of the active slots is widely adopted, and it is assumed that overlapping active slots will lead to mutual discovery [4,6,7,10,13]. However, the overlapping active slots may not be sufficient for mutual discovery, because the transceiver of a sensor node is half-duplex and there may exist collisions (two or more nodes transmit beacons at the same time). It is shown that, when the slot length is 10 ms, the 2-way discovery probability of 2-Beacon approach is lower than 50% (see Section 6.2). Third, the actual discovery latency of a time-slotted NDP depends not only on its theoretical discovery latency measured by the number of slots, but also on its slot length and discovery probability. Generally, the expected discovery latency of a time-slotted NDP is proportional to its theoretical discovery latency and the slot length, and inversely proportional to the discovery probability. Therefore, it is desired to increase the discovery probability while reducing the slot length. This task, however, is challenging because the slot length and the discovery probability are two conflicting factors, and they mainly depend on the beaconing strategy used by the NDP. The beaconing strategy refers to the mechanism for sending and receiving beacons, which is part of the NDP. The beaconing strategy has a significant impact on the actual performance of the NDP by changing the discovery probability and the slot length.

In this paper, we propose a new beaconing strategy, called Talk-Listen-Ack beaconing (TLA). We analyze the two-way discovery probabilities of TLA and 2-Beacon approach by using a fine-grained slot model. Further, we also analyze the two-way discovery probability of TLA that uses random backoff mechanism to avoid persistent collisions. Simulation and experimental results show that, compared with 2-Beacon approach, TLA can achieve a high discovery probability even in a short time slot. TLA is a generic beaconing strategy that can be applied to different slotted NDPs to reduce their expected discovery latency.

The remainder of this paper is organized as follows. Related works in the literature are discussed in Section 2. The motivation is given in Section 3. Talk-Listen-Ack Beaconing strategy is described in Section 4. Section 5 presents theoretical analysis of TLA and 2-Beacon approach. Evaluation of TLA and the comparison with 2-Beacon approach are provided in Section 6. The discussion is given in Section 7. Conclusion remarks are presented in Section 8.

## 2. Related Works

As a fundamental function of sensor networking, neighbor discovery has attracted much attention recently, and many energy-efficient NDPs have been proposed [1]. The proposed NDPs can be classified from different perspectives, such as slotted [3,4,5,6,7,8,10,11] or unslotted [12,14,15,16], probabilistic [3] or deterministic [3,4,5,6,7,8,9,10,11,15], single-channel [15] or multichannel [16,17,18], pairwise or collaborative [19], etc.

Many NDPs use time-slotted model where time is divided into time slots of equal length. A node generally repeats a cycle consisting of a certain number of time slots, where some slots are working (or active) slots, and other time slots are non-working slots. In an active slot, a node turns on the radio to send and/or receive beacons. The energy consumption of a slotted NDP is represented by duty cycle, which is the percentage of active slots in a cycle.

Time-slotted NDPs can be probabilistic or deterministic. As a representative of probabilistic NDPs, Birthday [3] transmits, listens, or sleeps with probabilities at each slot. Although Birthday has low average discovery latency, its worst-case discovery latency is unbounded. In contrast, the worst-case discovery latency of a deterministic NDP is bounded.

Disco [4], U-Connect [5], and Hello [7] are well-known representatives of deterministic NDPs. Coprime technique is used by Disco, where each node selects two primes, and by Chinese Remainder Theorem, there must exist overlapping active slots between two nodes. Besides coprime, U-Connect and Hello also adopt quorum technique [9] that guarantees overlapping active slots even if two nodes choose the same prime. All these three NDPs can be applied to the symmetric neighbor discovery where the duty cycles of two nodes are the same, and to the asymmetric neighbor discovery where nodes have different duty cycles.

Meng et al. [20] propose code-based schemes for symmetric and asymmetric neighbor discovery. The active-sleep pattern of a node is formulated as a 0–1 code. Based on the 0–1 code and set theory, the feasibility conditions for neighbor discovery are given. For symmetric neighbor discovery, the scheme called Diff-Codes is designed, which can achieve the tight worst-case latency bound when it can be extended from a perfect difference set. Diff-Codes is extended to ADiff-Codes designed for asymmetric neighbor discovery.

Disco uses a beaconing strategy that beacons are sent at the beginning and the end of the active slots so as to achieve mutual discovery when active slots of two nodes overlap. This 2-Beacon approach is widely accepted and adopted by other slotted NDPs [6,7,13]. Most slotted NDPs are designed with the assumption that overlapping active slots will lead to mutual discovery [4,6,7,10,13]. However, overlapping active slots combined with 2-Beacon approach cannot guarantee mutual discovery in real applications, which may lead to a large gap between the theoretical and real discovery performance.

Qiu et al. [8] point out it is sufficient to send one beacon in an active slot, and mutual discovery can be achieved through two separate one-way discoveries. Further, based on the observation that the transmission time of a beacon is much shorter than the slot length, a design principle called “Talk More Listen Less” is proposed, which aims to reduce the number of active slots by sending more beacons. However, the extra radio-on time for sending a beacon is ignored, which makes its real duty cycle larger than its nominal value since the extra radio-on time is much longer than the beacon’s transmission time in real systems [15]. Furthermore, neighbor discovery through two separate one-way discoveries is usually slower than direct two-way discovery in terms of discovery latency [15].

Existing deterministic slotted NDPs mostly use the number of slots instead of time units such as microseconds, milliseconds, or seconds to evaluate the discovery latency [12]. The actual discovery latency depends on several factors such as slot length, collisions, and energy consumption (duty cycle) [21,22,23,24]. Gu et al. propose a practical neighbor discovery framework with collisions, latency constraints, and energy consumption taken into account [24]. Bian et al. attempt to reduce the actual discovery latency by a fine-grained control over the slot length and the number of beacons sent [22]. Jin et al. investigate how the actual discovery latency can be reduced by simply reducing the slot length [21]. In our previous works [15,16], we, respectively, present generic analytical models for single- and multiple-channel neighbor discovery, in which the metric of discovery latency is units of time.

In short, for slotted NDPs, discovery latency is proportional to the slot length and inversely proportional to the discovery probability, and the slot length and the discovery probability are two conflicting factors. With the same goal of improving the actual discovery latency as the works [21,22,24], we study the beaconing strategy that can achieve a better tradeoff between the discovery probability and the slot length.

## 3. Motivation

Generally, for a slotted NDP, its expected discovery latency in time units, denoted by *X*, can be computed as:

(1) $$X=\frac{1}{P}\times N\times t_{slot},$$

where *P* is the discovery probability, *N* is the theoretical discovery latency in units of time slots, and $t_{slot}$ is the length of a time slot. In order to reduce the discovery latency *X*, we should increase *P* while reducing $t_{slot}$.

The discovery probability *P* depends on beaconing strategy used by the NDP and the slot length $t_{slot}$. 2-Beacon approach is widely adopted by most time-slotted NDPs, and it is assumed that overlapping active slots of two nodes leads to successful two-way neighbor discovery (i.e., P=1 in Equation (1)). This assumption, however, is too ideal to reflect the real performance. Next, we illustrate the reason by showing the detailed process of 2-Beacon approach based on a fine-grained time slot model.

**Figure 1:**
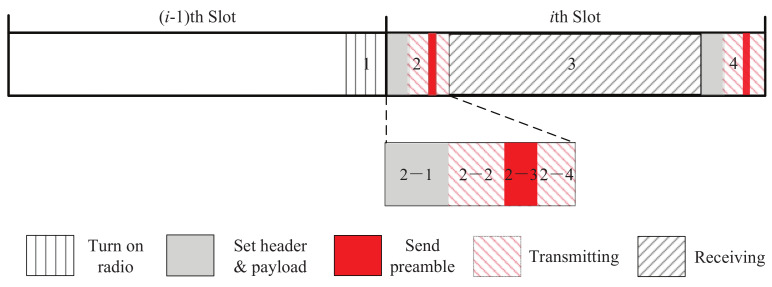
A fine-grained time slot model.

The detailed implementation of 2-Beacon approach is shown in Figure 1, where *i*th slot is an active slot, and (i−1)th slot is a non-working slot. The whole process is divided into four phases: turning on radio in advance (1), sending the first beacon (2), listening to the channel (3), and sending the second beacon (4). The durations of the four phases are denoted by $t_{\mathrm{radio}}$, $t_{b}$, $t_{\mathrm{rx}}$, and $t_{b}$, respectively. In the first phase, the node turns on the radio in advance in (i−1)th slot so that a beacon can be sent at the beginning of *i*th slot. Further, the second phase (or the fourth phase) is composed of four sub-phases: setting the header and payload of the beacon (2-1), loading the beacon into Tx buffer (2-2), sending synchronisation header (SHR) consisting of the preamble and Start of Frame Delimiter (SFD) (2-3), and sending the MAC protocol data unit (PDU) (2-4), as shown in Figure 1. The durations of these four sub-phases are denoted by $t_{\mathrm{hp}}$, $t_{\mathrm{load}}$, $t_{\mathrm{shr}}$, and $t_{\mathrm{pdu}}$, respectively. Let $t_{tx}$ represent the time when node is in transmitting state, we have $t_{tx}=t_{\mathrm{load}}+t_{\mathrm{shr}}+t_{\mathrm{pdu}}$, as shown in Figure 1.

Generally, it follows $t_{\mathrm{hp}}+t_{\mathrm{load}}>t_{\mathrm{shr}}+t_{\mathrm{pdu}}$. In other words, although the transmission time of SHR and PDU is short, the extra time (i.e., $t_{\mathrm{hp}}+t_{\mathrm{load}}$) spent before transmission is much longer than the transmission time. For instance, sending a 1-ms beacon needs extra two milliseconds. Furthermore, the transceiver of a sensor node is half-duplex, which means it cannot receive while sending a beacon. Specifically, when a node in transmitting state that includes 2-2, 2-3, and 2-4 in Figure 1, it cannot switch to receiving state before the sending is completed. As the length of listening window ($t_{\mathrm{rx}}$) becomes smaller, the discovery probability decreases. For instance, the discovery probability of 2-Beacon approach is no more than 50% when $t_{slot}=10$ ms (see Section 6.2).

Motivated by the above analysis, we design a new beaconing strategy, which can achieve a high discovery probability even in a short time slot. Compared with the 2-Beacon approach, the new beaconing strategy enables a slotted NDP to reduce its expected discovery latency.

## 4. Talk-Listen-Ack Beaconing

**Figure 2:**
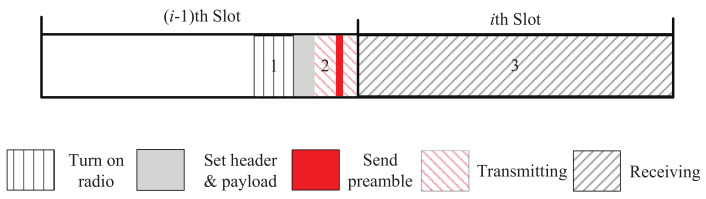
A time slot model of TLA.

In this section, we present our beaconing strategy, called Talk-Listen-Ack beaconing (TLA). The main idea of TLA is to reduce redundant beacons and save more time for receiving beacons. Figure 2 shows the time slot model of TLA, where *i*th slot is an active slot, and (i−1)th slot is a non-working slot. The active slot (*i*th slot) is dedicated to receiving neighbors’ beacons, and a beacon is sent at the end of previous slot ((i−1)th slot). This design aims to increase *P* and decrease $t_{slot}$ in Equation (1) so as to reduce the expected discovery latency.

**Figure 3:**
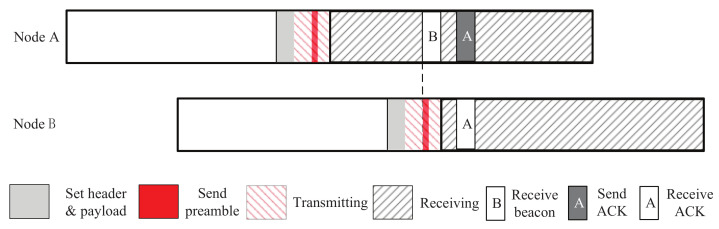
The discovery process of TLA.

Figure 3 shows the discovery process of TLA. When an active slot is arriving, node A first sends a beacon and then listens to the channel. Once a beacon is received from node B, node A will send an acknowledgment. The acknowledgement will be received by node B since it is listening the channel after sending its beacon. Thus, node A and B receive each other’s beacons and complete two-way discovery.

In order to respond quickly, TLA sends the acknowledgement by re-sending the beacon in Tx buffer. This can be done by changing the data sequence number (DSN) of the beacon in the Tx buffer and instructing the radio to send it immediately.

Compared with 2-Beacon approach, TLA has the following advantages. Firstly, TLA reduces the number of beacons sent by half. For each active slot, when no beacon from neighbors is received, TLA only sends one beacon. In addition, TLA can also accomplish two-way discovery: once a beacon is received from a neighbor, the node will respond with an acknowledgment so that the neighbor also discover the node. Secondly, TLA uses the beacon already in the Tx buffer to complete a quick response. By this method, the extra time cost of sending a beacon (i.e., $t_{\mathrm{hp}}$ and $t_{\mathrm{load}}$) is saved.

## 5. Performance Analysis of TLA Beaconing

### 5.1. Two-Way Discovery Probability

In this section, we analyze the two-way discovery probability of TLA and compare it with 2-Beacon approach. Here, the two-way discovery probability is defined as the probability that two nodes discover each other when their active time slots overlap. Consider a pair of overlapping active slots of two nodes, say node A and B. Denote $t_{offset}$ as the time difference between the beginning moment of sending the first beacon of node B and that of node A.

**Figure 4:**
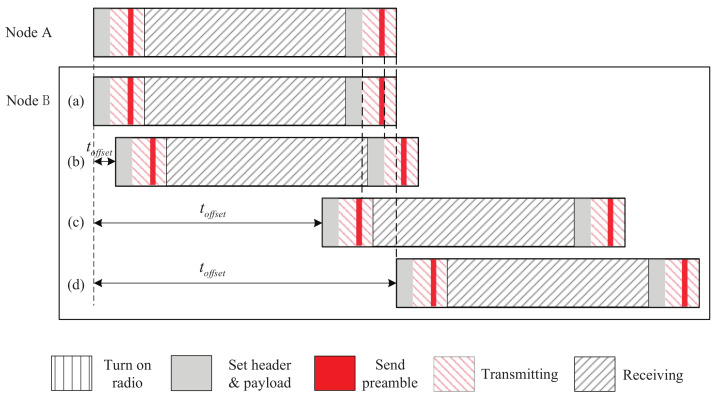
The discovery process of 2-Beacon approach as $t_{offset}$ changes.

We first analyze the two-way discovery probability of 2-Beacon approach. It is assumed that $t_{offset}$ is uniformly distributed over $\left[-t_{slot},t_{slot}\right]$. In addition, in the fined-grained slot model (presented in Section 3), we assume $t_{\mathrm{hp}}+t_{\mathrm{load}}>t_{\mathrm{shr}}+t_{\mathrm{pdu}}$ and $t_{\mathrm{pdu}}<t_{\mathrm{load}}$. Figure 4 shows the cases of 2-Beacon approach as $t_{offset}$ changes from 0 to $t_{slot}$. We have following cases:

1. When $t_{tx}-t_{pdu}\geq t_{offset}\geq 0$, that is, $t_{offset}$ varies from (a) to (b) shown in Figure 4, node B cannot receive the beacon from node A because node B’s radio is in transmitting state;
2. When $t_{slot}-t_{tx}-t_{b}+t_{pdu}\geq t_{offset}>t_{tx}-t_{pdu}$, i.e., $t_{offset}$ varies from (b) to (c) shown in Figure 4, node A and B can receive each other’s beacons, and thus, 2-way discovery occurs;
3. When $t_{slot}\geq t_{offset}>t_{slot}-t_{tx}-t_{b}+t_{pdu}$, i.e., $t_{offset}$ varies from (c) to (d) shown in Figure 4, node A cannot receive node B’s beacon.

The case when $t_{offset}$ changes from $-t_{slot}$ to 0 is symmetric with the above case. Overall, the two-way discovery probability of 2-Beacon approach, denoted by $P_{BB}$, is

(2) $$P_{BB}=\frac{t_{slot}-t_{b}-2t_{tx}+2t_{pdu}}{t_{slot}}.$$

**Figure 5:**
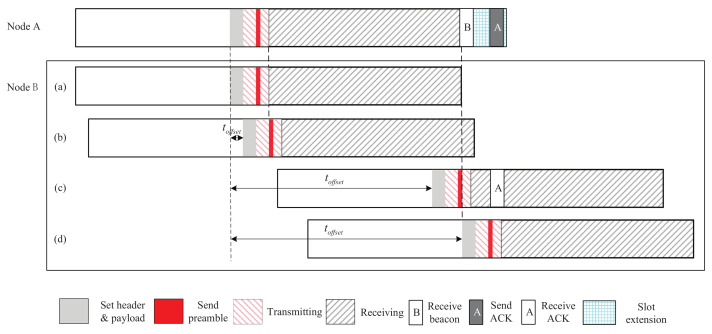
The discovery process of TLA as $t_{offset}$ changes.

For TLA, $t_{offset}$ is uniformly distributed over $\left[-t_{slot}-t_{b},t_{slot}+t_{b}\right]$. Figure 5 shows the cases of TLA when $t_{offset}$ ranges from 0 to $t_{slot}+t_{b}$. It follows that:

1. When $t_{shr}+t_{pdu}\geq t_{offset}\geq 0$, that is, $t_{offset}$ varies from (a) to (b) shown in Figure 5, node A cannot receive the beacon from node B and vice versa because they are all in transmitting state;
2. When $t_{slot}+t_{pdu}\geq t_{offset}>t_{shr}+t_{pdu}$, i.e., $t_{offset}$ varies from (b) to (c) shown in Figure 5, node A can receive the beacon from node B, and node B can receive the acknowledgement from node A. Note that, when node A receives the beacon from node B at the end of the active slot, it is allowed to complete the acknowledgement by slightly extending the active slot before going to sleep, as shown in Figure 5. Therefore, 2-way discovery occurs;
3. When $t_{slot}+t_{b}\geq t_{offset}>t_{slot}+t_{pdu}$, i.e., $t_{offset}$ varies from (c) to (d) shown in Figure 5, node A cannot receive node B’s beacon.

The case when $t_{offset}$ changes from $-t_{slot}-t_{b}$ to 0 is symmetric with the above case. Thus, the two-way discovery probability of TLA, denoted by $P_{TLA}$, is:

(3) $$P_{TLA}=\frac{t_{slot}-t_{shr}}{t_{slot}+t_{b}}.$$

### 5.2. Random Backoff

If two nodes transmit beacons at the same time, a collision occurs, leading to failure in discovery. For example, for the 2-Beacon approach, for the case (a) shown in Figure 4, there are collisions between the first pair and the second pair of beacons of node A and B. Furthermore, for TLA, for the case (a) shown in Figure 5, node A and B collide. Moreover, in the above cases, collisions will be persistent if $t_{offset}$ does not change when node A encounters node B the next time. To avoid persistent collisions, one feasible method is to adopt random backoff mechanism. Next, we present TLA strategy with random backoff mechanism, denoted by TLA-RB.

**Figure 6:**
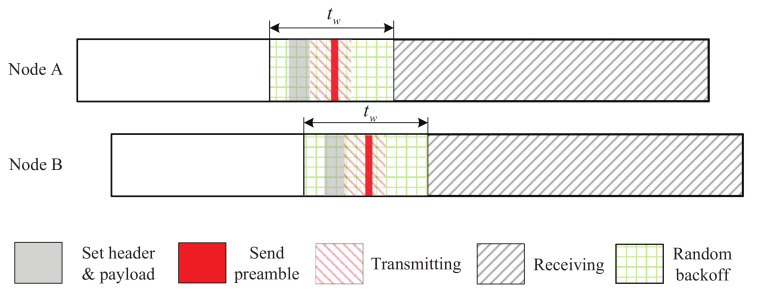
TLA strategy with random backoff mechanism.

As can be seen from Figure 6, in TLA-RB, the transmission of the beacon is contained in a window and the start time of the beacon is randomly selected within the window. The window is called beacon window. Next, we analyze the expected probability of two-way discovery of TLA-RB.

Consider the case where the beacon windows of node A and B overlap and the start time of the beacon window of node A is earlier than or equal to that of node B. Here, we take the start time of the beacon window of node A as the reference point. For TLA-RB, the time offset $t_{offset}$ is defined as the time difference of the start time of the beacon window of node B and that of node A. Further, the time difference between the time when the beacon of node A is sent and the start time of the beacon window of node A is called phase of node A, denoted by $\varphi_{A}$. Furthermore, the time difference between the time when the beacon of node B is sent and the start time of the beacon window of node A is called phase of node B, denoted by $\varphi_{B}$.

**Figure 7:**
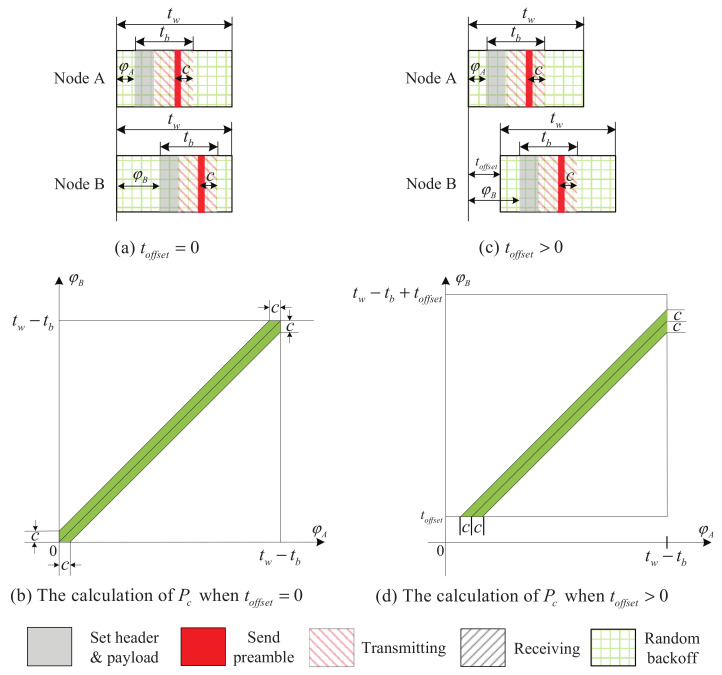
The overlapping pattern of two beacon windows and corresponding calculation of collision probability when $t_{w}>t_{b}+2c$.

Let $t_{w}$ be the length of beacon window, and $c=t_{shr}+t_{pdu}$. Given a value of $t_{offset}$, we can calculate the collision probability $P_{c}$ as follows. For presentation simplicity, in the sequel, we only discuss the case when $t_{w}>t_{b}+2c$, and the analysis of the other two cases, $t_{b}+c\geq t_{w}>t_{b}$ and $t_{b}+2c\geq t_{w}>t_{b}+c$, is similar. Figure 7a show the case when $t_{offset}=0$. In this case, it follows that $\varphi_{A}$ and $\varphi_{B}$ are independent and uniformly distributed in $\left[0,t_{w}-t_{b}\right]$. Furthermore, the probability of event $\left|\varphi_{B}-\varphi_{A}\right|\leq c$ (i.e., $P_{c}$) can be calculated as the ratio of the area of the shaded region shown in Figure 7b to ${\left(t_{w}-t_{b}\right)}^{2}$. When $t_{w}\geq t_{offset}>0$, as shown in Figure 7c, $\varphi_{A}$ is uniformly distributed in $\left[0,t_{w}-t_{b}\right]$, and $\varphi_{B}$ is uniformly distributed in $\left[t_{offset},t_{w}-t_{b}+t_{offset}\right]$. Imagine shifting the square of Figure 7b upward by $t_{offset}$ and extending the shaded area of Figure 7b, and we will have the resultant square and shaded area shown in Figure 7d. For $t_{offset}>0$, $P_{c}$ can be calculated as the ratio of the area of the shaded region shown in Figure 7d to ${\left(t_{w}-t_{b}\right)}^{2}$. Denote $P_{nc}$ the probability that there is no collision. It follows $P_{nc}=1-P_{c}$, and we have:

(4) $$p_{nc}=\left\{\begin{array}{cc} 1-\frac{2c}{t_{w}-t_{b}}+\frac{c^{2}+t_{offset}^{2}}{{\left(t_{w}-t_{b}\right)}^{2}} & \mathrm{if}\;c>t_{offset}>0;\\ 1-\frac{2c(t_{w}-t_{b}-t_{offset})}{{\left(t_{w}-t_{b}\right)}^{2}} & \mathrm{if}\;t_{w}-t_{b}-c>t_{offset}\geq c;\\ \frac{1}{2}+\frac{t_{offset}-c}{t_{w}-t_{b}}-\frac{{\left(t_{offset}-c\right)}^{2}}{2{\left(t_{w}-t_{b}\right)}^{2}} & \mathrm{if}\;t_{w}-t_{b}+c>t_{offset}\geq t_{w}-t_{b}-c;\\ 1 & \mathrm{if}\;t_{w}\geq t_{offset}\geq t_{w}-t_{b}+c. \end{array}\right.$$

**Figure 8:**
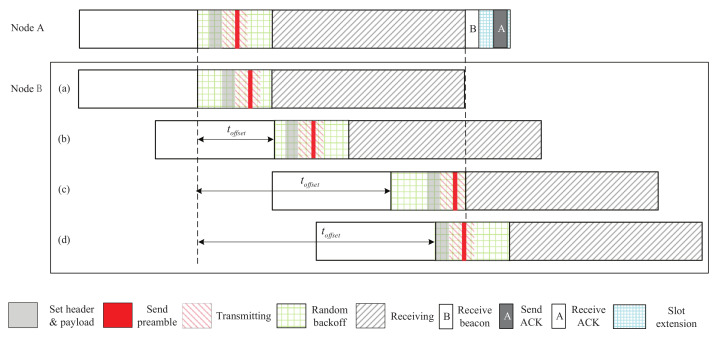
The discovery process of TLA-RB as $t_{offet}$ changes.

Denote $P_{TLA-RB}$ as the 2-way discovery probability of TLA-RB. Note that, given a $t_{offset}$, $P_{TLA-RB}$ is a random variable because of the backoff mechanism. So, we compute the expectation of $P_{TLA-RB}$, denoted by $E[P_{TLA-RB}]$. Figure 8 shows the discovery process of TLA-RB as $t_{offset}$ changes. 2-way discovery is possible only when $t_{offset}$ is from 0 to $t_{slot}+t_{w}-t_{hp}-t_{load}-t_{shr}$, as shown in Figure 8. Let $L=t_{slot}+t_{w}-t_{hp}-t_{load}-t_{shr}$. We divide *L* into three segments, $\left[0,t_{w}\right)$ (i.e., from (a) to (b) in Figure 8), $\left[t_{w},t_{slot}\right)$ (i.e., from (b) to (c) in Figure 8), and $\left[t_{slot},L\right]$ (from (c) to (d) in Figure 8). The expectation of 2-way discovery probability for the three segments are denoted by $E[P_{TLA-RB-I}]$, $E[P_{TLA-RB-II}]$, and $E[P_{TLA-RB-III}]$, respectively.

For the first segment, if there is no collision, then 2-way discovery can be achieved. Thus, we have $P_{TLA-RB}=p_{nc}$. By Equation (4), we have:

(5) $$\begin{array}{ccc} E[P_{TLA-RB-I}] & = & \frac{1}{L}\int_{0}^{c}\left(1-\frac{2c}{t_{w}-t_{b}}+\frac{c^{2}+t_{offset}^{2}}{{\left(t_{w}-t_{b}\right)}^{2}}\right)dt_{offset}\\ & + & \frac{1}{L}\int_{c}^{t_{w}-t_{b}-c}\left(1-\frac{2c(t_{w}-t_{b}-t_{offset})}{{\left(t_{w}-t_{b}\right)}^{2}}\right)dt_{offset}\\ & + & \frac{1}{L}\int_{t_{w}-t_{b}-c}^{t_{w}-t_{b}+c}\left(\frac{1}{2}+\frac{t_{offset}-c}{t_{w}-t_{b}}-\frac{{\left(t_{offset}-c\right)}^{2}}{2{\left(t_{w}-t_{b}\right)}^{2}}\right)dt_{offset}\\ & + & \frac{t_{b}-c}{L}\\ & = & \frac{t_{w}-c}{L}. \end{array}$$

For the second segment, we have:

(6) $$E\left[P_{TLA-RB-II}\right]=\frac{t_{slot}-t_{w}}{L}.$$

For the third segment, the two-way discovery probability is:

(7) $$P_{TLA-RB}=\left\{\begin{array}{cc} 1 & \mathrm{if}\;t_{slot}+t_{pdu}>t_{offset}\geq t_{slot};\\ \frac{L-t_{offset}}{t_{w}-t_{b}} & \mathrm{if}\;L\geq t_{offset}\geq t_{slot}+t_{pdu}. \end{array}\right.$$

Then, it follows:

(8) $$\begin{array}{ccc} E[P_{TLA-RB-III}] & = & \frac{t_{pdu}}{L}+\frac{1}{L}\int_{t_{slot}+t_{pdu}}^{L}\left(\frac{L-t_{offset}}{t_{w}-t_{b}}\right)dt_{offset}\\ & = & \frac{\frac{1}{2}\left(t_{w}-t_{b}\right)+t_{pdu}}{L}. \end{array}$$

Overall, we have:

(9) $$\begin{array}{ccc} E[P_{TLA-RB}] & = & E\left[P_{TLA-RB-I}\right]+E\left[P_{TLA-RB-II}\right]+E\left[P_{TLA-RB-III}\right]\\ & = & \frac{t_{slot}+\frac{1}{2}\left(t_{w}-t_{b}\right)-t_{shr}}{L}. \end{array}$$

For the case when $t_{b}+c\geq t_{w}>t_{b}$ or $t_{b}+2c\geq t_{w}>t_{b}+c$, we can similarly calculate $P_{c}$, $P_{nc}$, and $E[P_{TLA-RB}]$. The results of $E[P_{TLA-RB}]$ for these two cases are the same with Equation (9), and the details are omitted.

## 6. Evaluation

In this section, we compare TLA with 2-Beacon approach by simulations and experiments. The simulations were done by using MATLAB, and experiments were done in a testbed of TelosB motes.

### 6.1. Implementation

The testbed consists of a laptop and two TelosB motes, called node A and B. Nodes A and B are connected with the laptop through USB cables. The laptop is responsible for configuring the two motes and collecting results.

Based on the measurements, the values of parameters are as follows: $t_{b}=3$ ms, $t_{hp}=1$ ms, $t_{load}=1$ ms, $t_{shr}=0.2$ ms, and $t_{pdu}=0.8$ ms.

To compare TLA with 2-Beacon approach, we had implemented well-known slotted NDPs including Disco, U-Connect, and Hello on TinyOS 2.1.2. All these NDPs were implemented under the UPMA (Unified Radio Power Management Architecture) framework of TinyOS. For each NDP, we implemented two versions, one using TLA and the other using 2-Beacon approach. In the sequel, we use the name of a NDP followed by the name of a beaconing strategy to indicate a specific NDP. For instance, Disco that uses TLA is denoted as Disco-TLA, and Disco that uses 2-Beacon is denoted as Disco-2-Beacon.

In order to compare two beaconing strategies fairly, we should set them to have the same (or approximately the same) duty cycle. Assume a NDP using 2-Beacon repeats its active-sleep pattern every $n_{BB}$ slots, where $m_{BB}$ slots are active, and the slot length is $t_{slot-BB}$. Then, according to the slot model shown in Figure 1, its duty cycle, denoted by $DC_{BB}$, is $DC_{BB}=\frac{m_{BB}\;\times \;t_{slot-BB}}{n_{BB}\;\times \;t_{slot-BB}}=\frac{m_{BB}}{n_{BB}}$. For the NDP using TLA, assume its active-sleep pattern is repeated every $n_{TLA}$ slots, where $m_{TLA}$ slots are active, and the slot length is $t_{slot-TLA}$. Based on the slot model shown in Figure 2, the duty cycle of the NDP using TLA, denoted by $DC_{TLA}$, is $DC_{TLA}=\frac{m_{TLA}\;\times \;\left(t_{slot-TLA}\;+\;t_{b}\right)}{n_{TLA}\;\times \;t_{slot-TLA}}$.

Then, for a specific duty cycle value, we can select parameter values for the NDP using 2-Beacon and the NDP using TLA, respectively. We take Disco as an example. For the duty cycle of 5%, Disco-2-Beacon selects a pair of primes (37, 43) such that $DC_{BB}=\frac{37\;+\;43}{37\;\times \;43}\times 100\%\approx 5\%$. For the same duty cycle, assuming $t_{slot-TLA}$= 6 ms and $t_{b}$ = 3 ms, Disco-TLA chooses a pair of primes (53, 67), and it follows $DC_{TLA}=\frac{\left(53\;+\;67)\times (6\;+\;3\right)}{(53\;\times \;67)\;\times \;6}\times 100\%\approx 5\%$.

### 6.2. Simulation and Experimental Results

**Figure 9:**
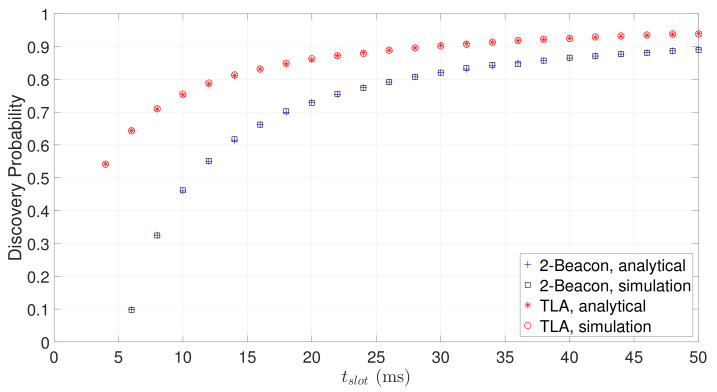
The discovery probability of TLA and 2-Beacon for different slot lengths.

Figure 9 shows the discovery probabilities of TLA and 2-Beacon against slot length. The analytical discovery probabilities of 2-Beacon and TLA are computed by Equations (2) and (3), respectively. It can be seen that the analytical results perfectly match with the simulated values, which verifies the accuracy of our analysis. As expected, the discovery probabilities of TLA and 2-Beacon increase as the slot length increases. TLA has a much higher discovery probability than 2-Beacon, especially when the slot length is short. When $t_{slot}=10$ ms, a typical value used by 2-Beacon approach, the discovery probability of 2-Beacon is no more than 50%, while the discovery probability of TLA is more than 70%. Further, for the same discovery probability, the slot length of TLA is much shorter than that of 2-Beacon. Therefore, compared with 2-Beacon, TLA has a smaller slot length and a higher discovery probability.

The expected discovery latency of a NDP is proportional to the slot length and inversely proportional to the discovery probability (see Equation (1)). Increasing the slot length can increase the discovery probability, but it may also increase the expected discovery latency. Therefore, for each beaconing strategy, there exists an optimal slot length that can achieve the lowest expected discovery latency. To verify this claim, we ran Disco-TLA and Disco-2-Beacon for symmetric duty cycle pairs (3%, 3%), (5%, 5%), and (7%, 7%), and asymmetric duty cycle pairs (1%, 5%), (1%, 10%), and (5%, 10%).

**Figure 10:**
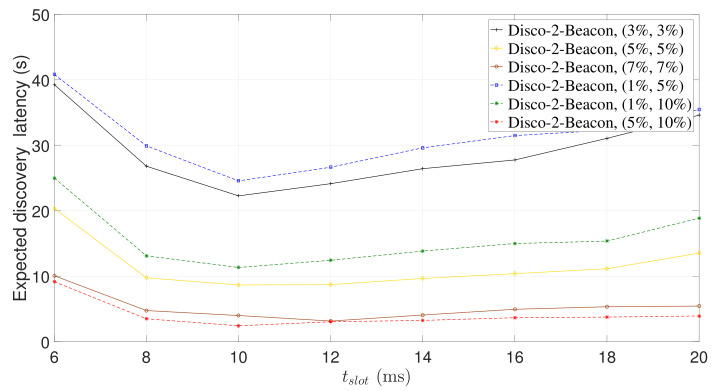
The expected discovery latency of Disco-2-Beacon for different slot lengths.

**Figure 11:**
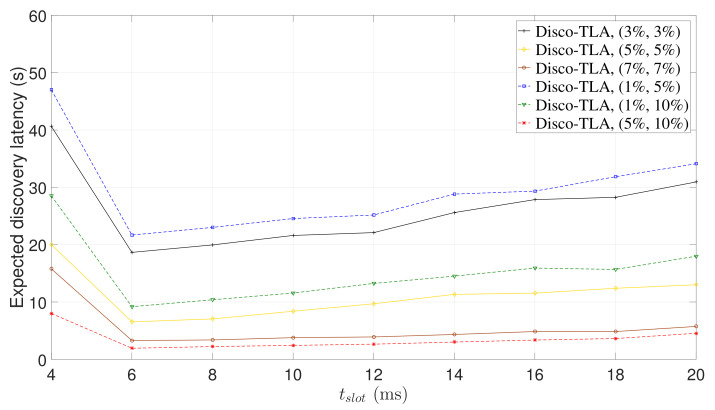
The expected discovery latency of Disco-TLA for different slot lengths.

Figure 10 and Figure 11 show the expected discovery latency of Disco-TLA and Disco-2-Beacon, respectively. The trend of the expected discovery latency validates our analysis. We observe that Disco-2-Beacon and Disco-TLA achieve the lowest expected discovery latency when $t_{slot}=10$ ms and $t_{slot}=6$ ms, respectively, as shown in Figure 10 and Figure 11. When $t_{slot}$ is smaller than 10 ms (or 6 ms), the expected discovery latency of Disco-2-Beacon (or Disco-TLA) will decrease as $t_{slot}$ increases due to the rapid increase in the discovery probability. However, when $t_{slot}$ is greater than 10 ms (or 6 ms), as $t_{slot}$ increases, the expected discovery latency of Disco-2-Beacon (or Disco-TLA) starts to increase because the increase of discovery probability slows down, and the expected discovery latency is dominated by the increase of slot length.

**Figure 12:**
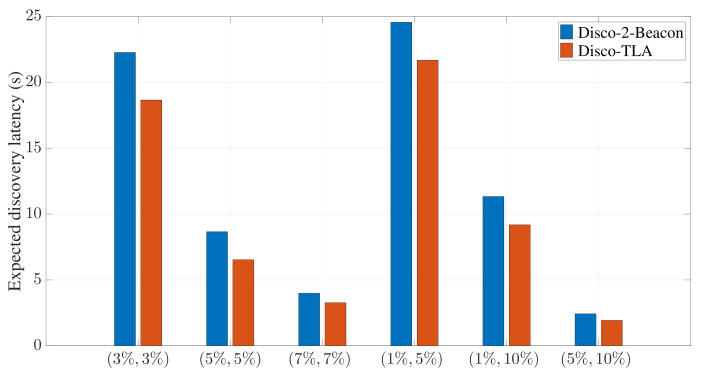
The expected discovery latency of Disco-TLA and Disco-2-Beacon for symmetric and asymmetric duty cycle pairs.

In the following evaluation, unless otherwise specified, $t_{slot}$ is set to 10 ms for 2-Beacon and 6 ms for TLA, respectively. Figure 12 provides a comparison between Disco-2-Beacon and Disco-TLA for multiple symmetric and asymmetric duty cycle pairs. Compared with Disco-2-Beacon, Disco-TLA reduces the expected discovery latency by 16.2%, 24.6%, 18.0%, 11.7%, 18.9%, and 20.5% for (3%, 3%), (5%, 5%), (7%, 7%), (1%, 5%), (1%, 10%), and (5%, 10%), respectively.

**Figure 13:**
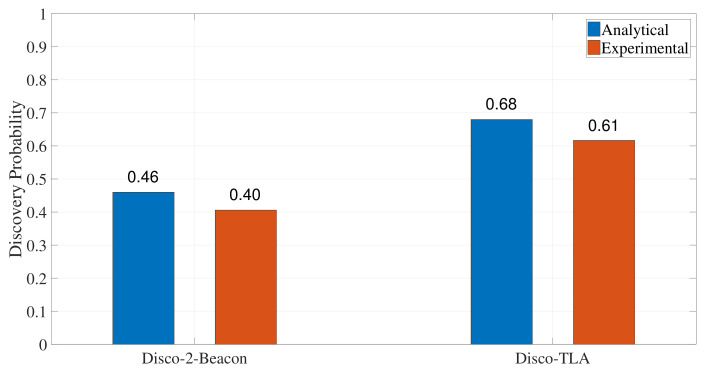
Discovery probabilities of Disco-TLA and Disco-2-Beacon.

Next, we compare TLA with 2-Beacon in a more comprehensive scenario. In this scenario, $DC_{A}$ and $DC_{B}$ are chosen from {1%,2%,⋯,10%}. There are 210=45 asymmetric duty cycle pairs, and 10 symmetric duty cycle pairs. 100 experiments were conducted for each duty cycle pair, and each experiment was run with a random time offset. Figure 13 gives the discovery probabilities of Disco-2-Beacon and Disco-TLA. The analytical discovery probabilities of Disco-2-Beacon and Disco-TLA are 0.46 and 0.68, while the experimental discovery probabilities of Disco-2-Beacon and Disco-TLA are 0.40 and 0.61, respectively. The slight decrease in experimental discovery probability is mainly due to interference from surrounding wireless devices. The experimental discovery probability of Disco-TLA is 21% greater than that of Disco-2-Beacon, and the slot length of Disco-TLA (i.e., 6 ms) is only three-fifths of that of Disco-2-Beacon (i.e., 10 ms).

**Figure 14:**
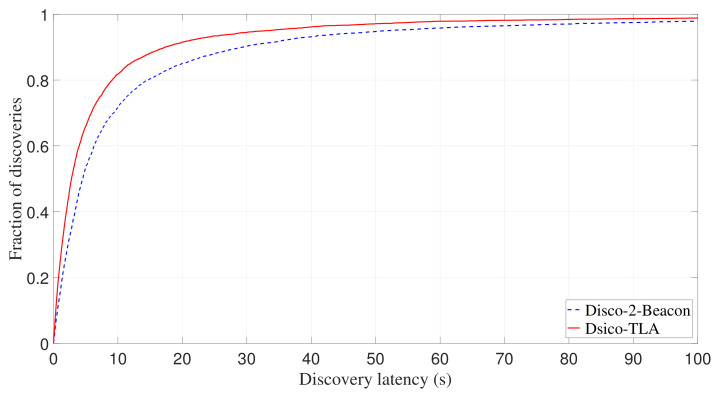
Experimental CDF of discovery latency of Disco-TLA and Disco-2-Beacon.

Figure 14 shows experimental cumulative distribution function (CDF) of discovery latency of Disco-2-Beacon and Disco-TLA. It can be observed that Disco-TLA performs much better than Disco-2-Beacon. Specifically, the average discovery latencies of Disco-2-Beacon and Disco-TLA are 13.78 s and 9.82 s, respectively, and compared with Disco-2-Beacon, Disco-TLA reduces the average discovery latency by 28.7%.

**Figure 15:**
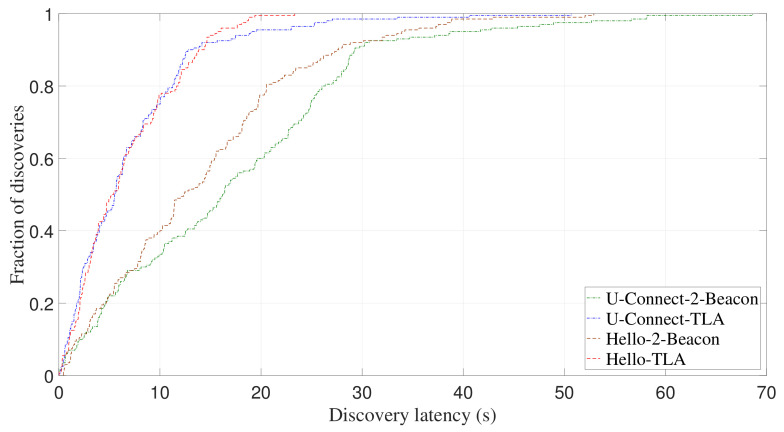
Experimental CDF of discovery latency of U-Connect and Hello for asymmetric duty cycle pair (1%, 10%).

**Figure 16:**
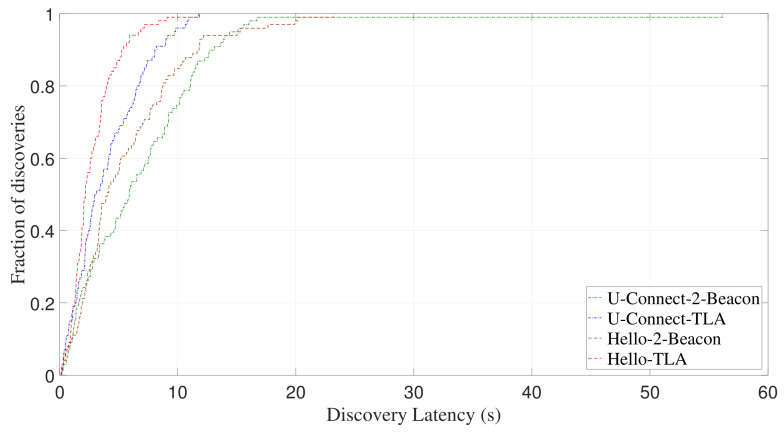
Experimental CDF of discovery latency of U-Connect and Hello for asymmetric duty cycle pair (3%, 7%).

Besides Disco, we also compare the performance of other NDPs using 2-Beacon and TLA, such as U-Connect and Hello. Figure 15 and Figure 16 show CDF of discovery latency of U-Connect and Hello for asymmetric duty cycle pairs (1%, 10%) and (3%, 7%), respectively. It can be seen that, U-Connect or Hello using TLA can significantly reduce their discovery latency. Specifically, for (1%, 10%) and (3%, 7%), U-Connect-TLA reduces the average discovery latency of U-Connect-2-Beacon by 59.1% and 42.2%, respectively, and Hello-TLA reduces the average discovery latency of Hello-2-Beacon by 53.4% and 50.3%, respectively.

**Figure 17:**
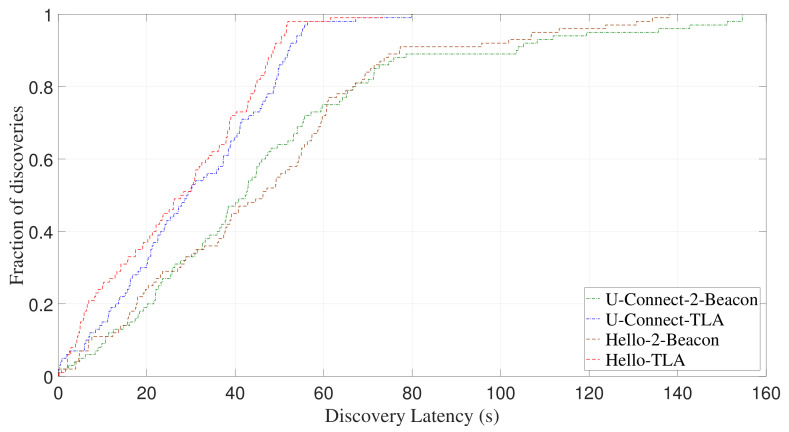
Experimental CDF of discovery latency of U-Connect and Hello for symmetric duty cycle pair (2%, 2%).

**Figure 18:**
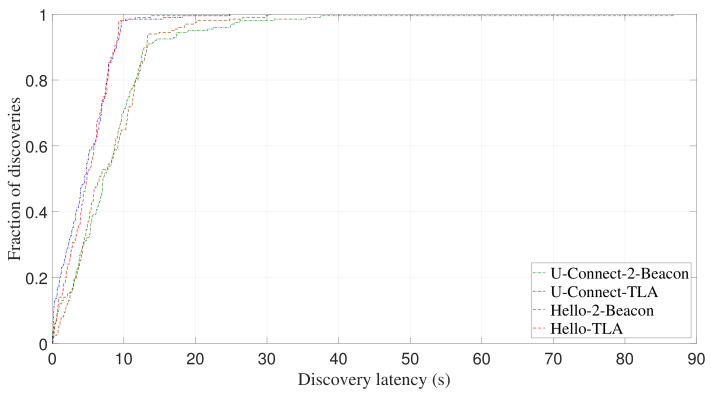
Experimental CDF of discovery latency of U-Connect and Hello for symmetric duty cycle pair (5%, 5%).

Figure 17 and Figure 18 show CDF of discovery latency of U-Connect and Hello for symmetric duty cycle pairs (2%, 2%) and (5%, 5%), respectively. Similar to the case of asymmetric duty cycle, U-Connect and Hello benefit from the use of TLA to reduce the discovery latency. More specifically, for (2%, 2%) and (5%, 5%), U-Connect-TLA reduces the average discovery latency of U-Connect-2-Beacon by 36.3% and 43.7%, respectively, and Hello-TLA reduces the average discovery latency of Hello-2-Beacon by 41.8% and 36.5%, respectively.

In addition to the average discovery latency, TLA also significantly reduces the worst-case discovery latency of U-Connect and Hello. For example, when TLA is adopted, the worst-case discovery latency of U-Connect is reduced by 71.4% for (5%, 5%) as shown in Figure 18, and the worst-case discovery latency of Hello is reduced by 55.9% for (1%, 10%) as shown in Figure 15. All these results demonstrate that TLA can be applied to different slotted NDPs to improve their discovery performance.

**Figure 19:**
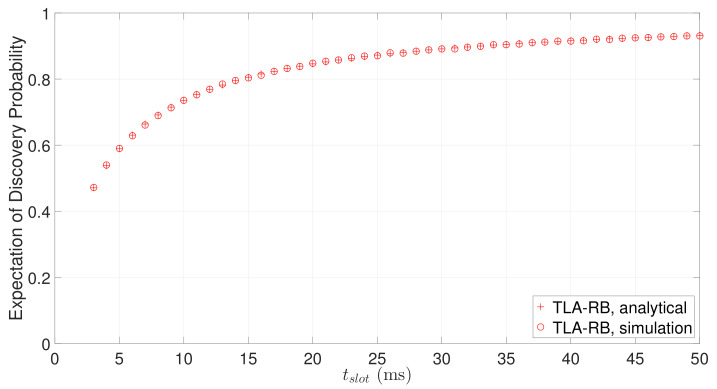
The expectation of discovery probability of TLA-RB for different slot lengths with $t_{w}=4$ ms.

**Figure 20:**
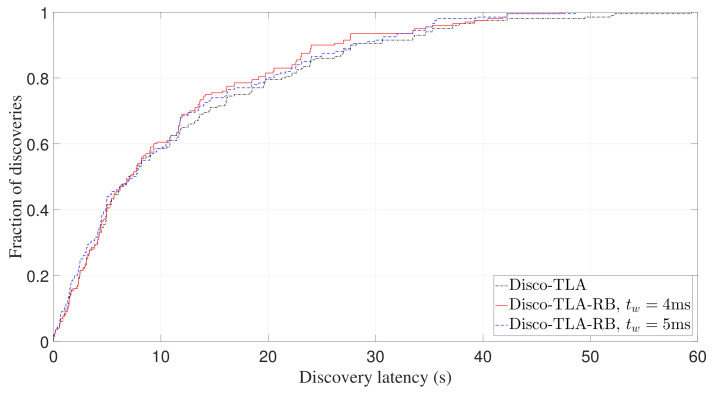
Experimental CDF of discovery latency of Disco-TLA-RB for asymmetric duty cycle pair (1%, 10%).

**Figure 21:**
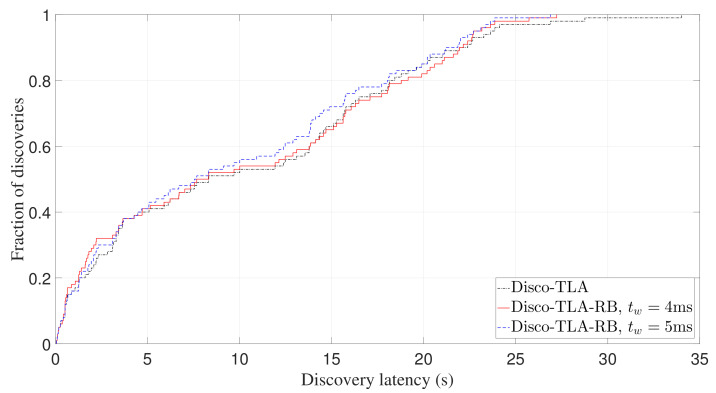
Experimental CDF of discovery latency of Disco-TLA-RB for symmetric duty cycle pair (5%, 5%).

Finally, we study whether TLA-RB can effectively avoid collisions. As can be seen from Figure 14, more than 95% discoveries are within 60s for both two beaconing strategies. The latency for the remaining 5% discoveries ranges from 60s to 100s, and this long tail distribution is mainly caused by collisions. TLA-RB aims to avoid collision by using backoff mechanism. Figure 19 shows the expectation of discovery probability of TLA-RB against slot length with $t_{w}$ being 4 ms. It can be seen that the simulation results match well with the analytical results (computed by Equation (9)), which verifies the accuracy of our analysis. Further, we conducted experiments of Disco using TLA-RB, with $t_{w}$ being 4 ms and 5 ms, respectively, and compared with Disco-TLA. Figure 20 and Figure 21, respectively, show CDF of discovery latency of Disco for an asymmetric duty cycle pair (1%, 10%) and a symmetric duty cycle pair (5%, 5%). As can be seen from Figure 20 and Figure 21, compared to TLA, TLA-RB can reduce the worst-case discovery latency, which means it can effectively avoid collisions. Note that although increasing $t_{w}$ can better avoid collisions, it will also increase $t_{slot}$ if the duty cycle remains the same, which in turn may increase the discovery latency. So, $t_{w}$ should be set to an appropriate value. It can be seen that, TLA-RB with $t_{w}=4$ ms has balanced performance for symmetric and asymmetric discovery, as shown in Figure 20 and Figure 21.

## 7. Discussion

In this section, we discuss more about ideas or methods that may improve the efficiency of a beaconing strategy. The beaconing strategy is part of a NDP and is responsible for sending and receiving beacons. Recall that the expected discovery latency of a NDP is $X=\frac{1}{P}\times N\times t_{slot}$ (Equation (1)). Although it cannot determine the theoretical performance of a NDP (i.e., *N*), the beaconing strategy has a significant impact on the actual performance of the NDP by changing the discovery probability (i.e., *P*) and the slot length (i.e., $t_{slot}$).

2-Beacon integrates beacon sending and receiving into an active time slot. In contrast, Nihao [8] and BlindDate [13] use another beaconing strategy that beacon is not placed in the active slot. This design is based on the idea that the beacon is much shorter than the active slot, and sending more beacons can reduce the number of active slots. However, based on the fine-grained slot model, we show that the extra cost of sending a beacon is not negligible. For example, sending a 1-ms beacon needs extra two milliseconds. In fact, placing beacons outside of the active slot can increase the discovery probability because the active slot is dedicated to receiving beacons. Compared with the method used by Nihao, TLA also separates beacon from active slot, but intends to send fewer rather than more beacons. In addition, unlike Nihao using two separate one-way discoveries to achieve mutual discovery, TLA uses acknowledgments to realize mutual discovery, thereby improving the discovery performance.

Diff-Codes or ADiff-Codes [20] use another beaconing strategy that active slots are overflowed so as to leverage non-alignment of slot boundaries to reduce the discovery latency. Specifically, an active slot will start δ time units earlier to send a beacon. The design of Diff-Codes or ADiff-Codes ensures that the overlapping active slots of two nodes is at least δ, which results in successful discovery, especially when δ is much larger than the beacon’s transmission time. Compared with the method used by Diff-Codes or ADiff-Codes, TLA also places the beacon transmission in the preceding slot of an active slot. The difference is that TLA does not send another beacon (or an acknowledgment) in the active slot unless a beacon is received from a neighboring node.

Collision is another factor that should be considered when designing a beaconing strategy. When the active-sleep patterns of two nodes are synchronized, it may cause persistent collisions between the two nodes, resulting in the worst-case discovery latency much larger than the theoretical value. Methods for mitigating collisions include reducing the number of beacons [21], dynamically adjusting slot length [22], and introducing randomization [21]. Compared with 2-Beacon, TLA reduces the number of beacons by half. In addition, TLA-RB uses random backoff mechanism to mitigate collisions.

Most slotted NDPs rely on overlapping active slots to realize neighbor discovery. In contrast, unslotted NDPs [14,15,16] explicitly require that a beacon from one node must fall into the listening window of another node to guarantee successful discovery. In addition, the discovery latency is measured by the unit of time rather than the number of time slots. However, like slotted NDPs, unslotted NDPs are also susceptible to collisions, so the beaconing strategy can use randomization to alleviate collisions. For example, Bluetooth low energy (BLE) adds a pseudo-random delay before sending advertisements (i.e., beacons) [16].

## 8. Conclusions

In this paper, we present talk-listen-ack beaconing strategy that can be applied to different slotted neighbor discovery protocols to reduce their expected discovery latency. TLA uses four mechanisms to improve its efficiency. First, the active slot is dedicated to receiving beacons to increase the discovery probability. Second, only one beacon is sent in the preceding slot of the active slot, and compared with 2-Beacon, the number of beacons is reduced by half. Third, once a beacon is received from a neighbor, an acknowledgment is sent to realize mutual discovery. Fourth, add a random delay before sending the beacon to mitigate collisions. Simulation and experimental results show that, compared with 2-Beacon approach, TLA can achieve a high discovery probability even in a short time slot.

The beaconing strategy has a significant impact on the actual performance of the neighbor discovery protocols. Future research topics include designing efficient beaconing strategies for different wireless technologies, such as ZigBee, WiFi, BLE, etc, and the design needs to consider the different characteristics of wireless technologies. Future research topics also include designing efficient beaconing strategies for different types of neighbor discovery protocols, such as unslotted neighbor discovery and collaborative neighbor discovery.

## Data Availability

Not applicable.
